# Giant acceleration of polaron transport by ultrafast laser-induced coherent phonons

**DOI:** 10.1126/sciadv.adg3833

**Published:** 2023-08-16

**Authors:** Hui-Min Wang, Xin-Bao Liu, Shi-Qi Hu, Da-Qiang Chen, Qing Chen, Cui Zhang, Meng-Xue Guan, Sheng Meng

**Affiliations:** ^1^ Beijing National Laboratory for Condensed Matter Physics and Institute of Physics, Chinese Academy of Sciences, Beijing 100190, China.; ^2^ School of Physical Sciences, University of Chinese Academy of Sciences, Beijing 100190, China.; ^3^ Songshan Lake Materials Laboratory, Dongguan, Guangdong 523808, China.; ^4^ Centre for Quantum Physics, Key Laboratory of Advanced Optoelectronic Quantum Architecture and Measurement (Ministry of Education), School of Physics, Beijing Institute of Technology, Beijing 100081, China.

## Abstract

Polaron formation is ubiquitous in polarized materials, but severely hampers carrier transport for which effective controlling methods are urgently needed. Here, we show that laser-controlled coherent phonon excitation enables orders of magnitude enhancement of carrier mobility via accelerating polaron transport in a prototypical material, lithium peroxide (Li_2_O_2_). The selective excitation of specific phonon modes, whose vibrational pattern directly overlap with the polaronic lattice deformation, can remarkably reduce the energy barrier for polaron hopping. The strong nonadiabatic couplings between the electronic and ionic subsystem play a key role in triggering the migration of polaron, via promoting phonon-phonon scattering in *q* space within sub-picoseconds. These results extend our understanding of polaron transport dynamics to the nonequilibrium regime and allow for optoelectronic devices with ultrahigh on-off ratio and ultrafast responsibility, competitive with those of state-of-the-art devices fabricated based on free electron transport.

## Teaser

Light-controlled coherent phonon excitation enables orders of magnitude enhancement in polaron mobility.

## INTRODUCTION

Polaron, as a quasiparticle composed of excess carrier dressed by a cloud of virtual phonons (*1*–*3*), intrinsically exists in materials upon charge doping due to strong electron-phonon interactions (EPIs) (*4*–*16*). It plays a central role in determining multifarious physicochemical properties including superconductivity (*3*, *17*, *18*), thermoelectricity (*4*), and photocatalysis (*19*–*21*). However, the carrier mobility (μ) is also greatly reduced owing to the concurrent propagation of surrounding lattice distortions when polarons are formed, severely hampering the practical applications of associated materials in electronic devices (*1*, *2*, *5*, *6*). For example, metal oxides are widely applied in energy conversion and optoelectronic devices, but the strong polarization or deformation of lattice (e.g., the metal-oxide polyhedron) facilitates the formation of small polarons, where excess carriers are self-trapped in a spatial range comparable to lattice constants (*11*–*13*, *22*). The carrier transport can thus switch from band transport of free carriers with mobility μ > 10 cm^2^/V · s to inconsecutive hopping of small polarons with μ < 0.1 cm^2^/V · s (*12*, *22*–*25*). One of such prototypical cases is in lithium-air battery; the low electrical conductivity and large overpotential were attributed to polaron formation in lithium peroxide (Li_2_O_2_), a prevailing intermediate under battery operations (*12*, *26*).

Strategies utilized to accelerate polaron transport are urgently needed to overcome the above limitations. However, the only feasible method to date is elevating temperature as more polaron hopping channels could be provided by larger lattice distortions, yet in fact, it is inefficient and severely limited by the operating temperature of devices (*1*, *2*). Recently, manipulating the dynamics of (quasi)particles via ultrafast laser excitation has become one of the most efficient approaches for controlling macroscopic properties of materials (*27*, *28*). Ultrafast spectroscopies have been used to monitor polaron dynamics, whereas most of them are limited to characterize polaron formation process. Only speculations about polaron transport mechanisms at the macroscopic scale were presented (*4*, *5*, *9*, *11*, *29*–*34*). Utilizing infrared light to accelerate polaron transport has been reported recently, which just relies on the heating effect instead of intrinsically improving polaron mobility (*35*). Advanced theoretical modeling with first-principles density functional theory (DFT) (*22*, *23*, *36*–*38*) and molecular dynamics (MD) simulations (*15*, *39*–*41*) could play an indispensable role in unveiling these conundrums. However, most studies rely on adiabatic approximations and only focus on static and thermodynamic properties of polarons, incapable of describing the real-time polaron dynamics with strong EPI.

In this work, we show that ultrafast laser pulses could accelerate polaron transport by orders of magnitude in a prototype metal-oxide Li_2_O_2_. The coherent excitation of specific vibrational modes (e.g., transverse optical modes *TO1* and *TO2*), whose vibrational pattern largely overlap with polaronic lattice deformation, can notably reduce the energy barrier for polaron hopping. Strong scattering among phonons from *q* = 0 to *q* ≠ 0 is found within sub-picoseconds due to strong nonadiabatic (NA) couplings between electrons and phonons, which is vital to trigger the propagation of polaronic lattice deformations. Our results reveal the key role of coherent phonon excitation in promoting polaron transport, and thus carrier mobility can be increased by eight orders of magnitude via tuning phonon amplitude utilizing different laser parameters (e.g., wavelength and intensity). The results provide important insights into the nonequilibrium dynamics of polarons and offer an efficient strategy for ultrafast control of polaron transport in materials.

## RESULTS

**Fig. 1.**
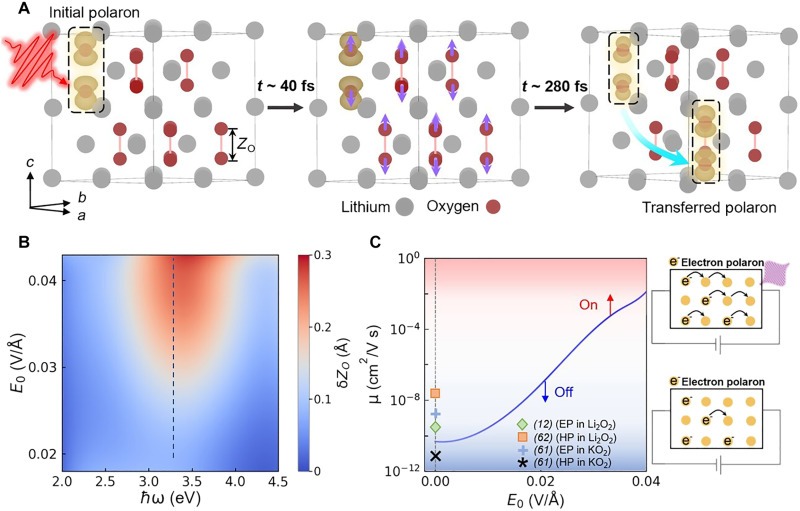
Laser-controlled polaron transfer and its potential applications. (**A**) Schematics of polaron transfer dynamics in Li_2_O_2_ upon photoexcitation. The initial polaron (IP) (left), coherent phonon excitations (middle), and transferred polaron (TP) (right) are shown. The purple and cyan arrows are guides for atomic displacements along *TO1@*Γ mode and polaron transfer direction, respectively. The bond length of an O-O dimer (OD) is labeled as *Z_O_*, and its variation is δ*Z_O_*. The charge density of polaron is plotted as yellow clouds at the isosurface of 7 × 10^−2^ e/Å^3^. (**B**) Phase diagram of *TO1@*Γ mode excitation as a function of photon energy ℏω and laser peak amplitude *E*_0_. The blue dashed line labels the case of ℏω = 3.3 eV. (**C**) Optical controlled electron mobility (μ) increasing exponentially with varied *E*_0_ at ℏω = 3.3 eV, which is calculated based on the Einstein relation (*60*) considering modified hopping energy barrier (note S5). The case under equilibrium condition (*E*_0_ = 0 V/Å) is compared with available data in literature involving electron (EP) or hole polarons (HP) in Li_2_O_2_ and its analogues, all showing the ultralow mobility (*12*, *61*, *62*). The upper and lower schematic diagrams depict the fast conductive (on) and blocked (off) state of the electrical device with and without appropriate laser irradiation, respectively.

The formation and transport dynamics of polaron in Li_2_O_2_ are shown in Fig. 1A. Before the laser illumination (*t* = 0 fs), the doping concentration with an excess electron per 2 × 2 × 1 Li_2_O_2_ unit cells (~3.3 × 10^21^ cm^−3^) can suppress the potential Mott transition (*36*, *42*). The stably formed polaron shows an additional electron trapped around one of the O-O dimers (ODs), leading to the out-of-plane (along the *c* axis) stretch and cleavage of the OD, which is referred to as the initial polaron (IP) state (*12*, *37*). To validate the model, we have tested a larger supercell containing 4 × 4 × 2 Li_2_O_2_ unit cells, to check that the main characteristics of polaron, e.g., the charge density distribution of excess electrons and the lattice distortions, are identical in both simulations (see note S2 and figs. S1 and S2).

To trigger polaron transport, linearly polarized laser pulses are applied with a time-dependent electric field waveform *E*(*t*) = *E*_0_ cos (ω*t*) exp [−(*t* − *t*_0_)^2^/2σ^2^] along the crystalline *b* axis. The typical photon energy ℏω, width σ, and peak amplitude of electric field *E*_0_ are set as 2.0 eV, 4 fs, and 0.036 V/Å, respectively (fig. S5). Upon laser irradiation, coherent lattice vibrations, particularly the synchronized out-of-plane stretch of ODs, are excited (*t =* 40 fs), which eventually leads to the migration of polaronic lattice deformation, i.e., the concurrent recovery of the stretched OD at the initial site and the OD bond stretch (by ~0.6 Å) at another site. After ~280 fs, most of the trapped excess electrons have been redistributed around the newly generated polaron, which is referred to as the transferred polaron (TP) state.

**Fig. 2.**
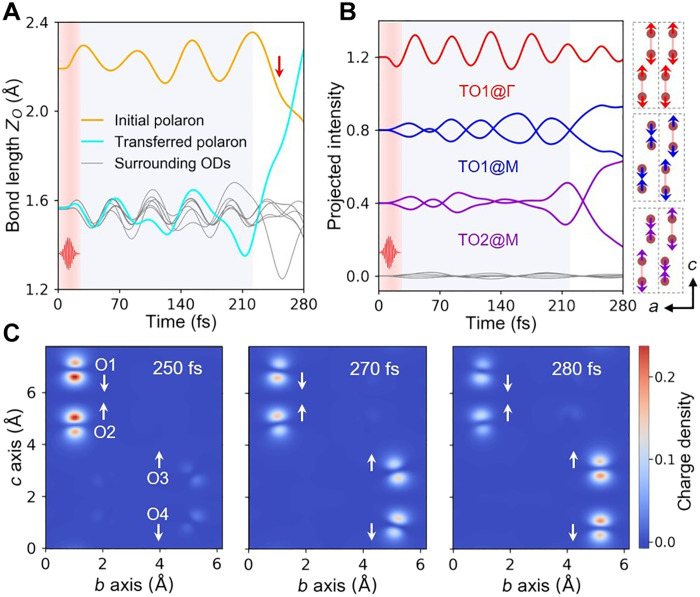
The nonequilibrium dynamics of polaron transfer. (**A**) Time evolution of *Z_O_*, which are divided into stage I (25 to 220 fs, gray shadow region) and stage II (220 to 280 fs). The red arrow labels the moment when self-trapped electron starts to transfer (250 fs). (**B**) The projected intensity of atomic displacements onto vibrational eigenmodes, where the plots have been shifted vertically for clarity. For the threefold degenerate *TO1@M* and *TO2@M* modes, only two of them with considerable intensity are plotted. The gray thin lines represent vibrational modes with negligible contribution. The eigenvectors of three dominant modes are shown, where the dotted boxes label the primitive cell and only the oxygen atoms are displayed. (**C**) Transfer dynamics of excess electron from the initial trapping site (O1-O2 dimer) to the new site (O3-O4 dimer). The white arrows are used to mark the moving direction of oxygen atoms along the *c* axis.

To investigate the mechanism of laser-driven polaron transport, time evolution of lattice geometry and charge density are analyzed. The structural distortion is characterized by the variation in OD bond length (*Z_O_*), which can be divided into two substages (Fig. 2A). In the first stage (25 to 220 fs), after the end of laser pulse (25 fs), all the ODs oscillate around the equilibrium position, indicating the excitation of coherent phonon modes. In the second stage (220 to 280 fs), while the oscillation of surrounding ODs irrelevant to polaron transfer is rather robust; *Z_O_* of initial (transferred) polarons continuously decreases (increases), leading to the recovered OD with *Z_O_ <* 2.0 Å (the broken OD with *Z_O_* > 2.0 Å) at their respective sites. When the ODs at the TP are stretched far away from equilibrium positions (250 fs), polaronic charge density comprising the anti-bonding states of OD is decreased (increased) at IP (TP), which, in turn, facilitates the out-of-plane contraction (stretch) of the OD (Fig. 2C). Therefore, complete polaron transfer is achieved via mutual promotion between the migration of lattice distortion and charge transport. The featured dynamic behaviors in the two substages also reveal that excitation and scattering of phonon modes might play an essential role in the laser-enhanced polaron transport.

To distinguish the dominant phonon modes contributing to the ultrafast dynamics, atomic displacement vectors are projected onto vibrational eigenmodes of the primitive cell with both zero (i.e., Γ point) and nonzero (e.g., *M* point) wave vectors considered (Fig. 2B and fig. S9, A and B). In the first stage, a zone-center transverse optical mode, i.e., *TO1@*Γ mode, is predominantly excited, attributed to the strongest EPI between it and photoexcited band-edge carriers (note S4). Meanwhile, *TO* modes with the nonzero wave vector, i.e., *TO1@M* and *TO2@M* modes, start to oscillate but with smaller vibrational amplitudes. Different from the synchronized out-of-plane stretch of all ODs along *TO1@*Γ mode in the first stage, *TO1* and *TO2* modes with $q=\vec{M}$ characterize the stretch of neighboring ODs in adjacent primitive cells in the opposite direction. *TO2* mode additionally features in the stretch of interlayer ODs in the opposite direction (see schematics in Fig. 2B). In the second stage, rapid and strong phonon-phonon scatterings from *TO1@*Γ to *TO1@M* and *TO2@M* modes occur, accounting for different variations of *Z_O_* between IP and TP (Fig. 2A).

**Fig. 3.**
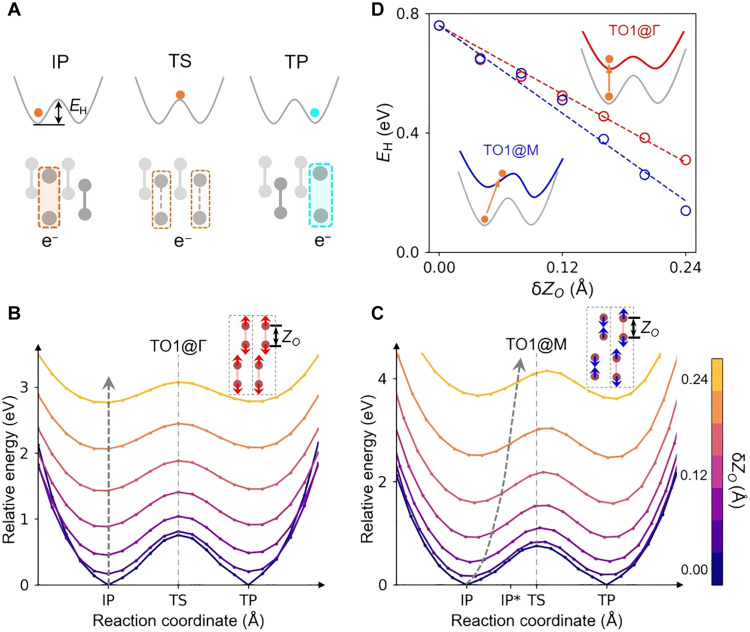
Effects of coherent phonon excitation on polaron transport. (**A**) PES along the polaron hopping pathway from IP to TP. The energy difference between IP and transition state (TS) is *E*_H_. (**B** and **C**) Modulated PES due to excitation of *TO1@*Γ and *TO1@M* modes with different amplitudes of δ*Z_O_*. The gray dashed lines show the modified position of the initial polaron (i.e., IP*) along a hopping trajectory with increased phonon amplitude. The reaction coordinate of PES is defined by the bond length difference between the ODs where the IP and TP are located. The excited phonon strength is described by the induced bond length variation of an OD (δ*Z_O_*). (**D**) Dependence of *E*_H_ on δ*Z_O_*, where circles are calculation data and dashed lines are linear fit. The insets schematically depict the PES with and without phonon excitations.

The effect of coherent atomic vibrations on polaron transport can be understood by means of potential energy changes, as shown in Fig. 3. On the basis of Marcus theory, a double-well potential energy surface (PES) is conventionally used to describe polaron transfer under thermal equilibrium conditions (*12*, *23*, *24*). The atomic structures along polaron transfer pathway are obtained by the linear interpolation of geometries between the IP and TP states (*24*). Two potential energy minimums correspond to the atomic configurations where the polaron is localized at initial (IP) or transferred (TP) lattice sites. To achieve polaron transfer, the system needs to overcome a hopping energy barrier (*E*_H_), and the polaron is evenly shared by the two sites when a transition state (TS) is reached (Fig. 3A). To reveal the relative role of different phonon modes in polaron transfer, phonon-modulated PES are constructed with atomic configurations generated by superposing the interpolated structures at thermal equilibrium with atomic displacements along the eigenvector of a certain phonon mode. The corresponding evolution of PES is shown in Fig. 3 (B and C) and figs. S7 and S8.

The common role of three dominant coherent phonons is to reduce *E*_H_ (see Fig. 3, B and C, and fig. S7A). The decrease of *E*_H_ is linearly dependent on the phonon amplitude (Fig. 3D and fig. S7B), which leads to an exponential increase of polaron hopping possibility (note S5). In contrast, the contribution of those modes associated with the vibrations of lithium atoms or the shear motions of oxygen atoms is negligible (fig. S8). Therefore, we confirm that only vibrational modes whose eigenvectors directly overlap with the pattern of polaronic lattice distortions (i.e., the out-of-plane elongation of ODs) contribute to the acceleration of polaron transport in Li_2_O_2_.

Compared to the zone-center phonon modes, the *TO1* and *TO2* modes with nonzero wave vectors can additionally trigger the transport of polaronic lattice deformation. Taking the *TO1@M* mode as an example, the opposing variation of *Z_O_* between adjacent primitive cells facilitates the migration of polaron along this hopping pathway. With the enhanced excitation of *TO1@M* mode, the lattice structure deviates away from that at potential energy minimum, but becomes close to that of the transition state (Fig. 3C). Meanwhile, the PES is tilted toward one of potential wells, so that the transition from TS to TP is more energetically favorable, whereas excitation of *TO1@*Γ mode characterizes the in-phase motions among atoms and fails to modify the localized lattice features (Fig. 3B). Therefore, mode conversion from the predominantly photoexcited *TO1@*Γ mode to the *TO1@M* and *TO2@M* modes is of great importance, which determines polaron transfer rate. To our surprise, the phonon-phonon scattering from *TO1@*Γ to the *TO1* and *TO2* modes with nonzero wave vectors take place within hundreds of femtoseconds, much faster than that for conventional lattice relaxation (*43*–*45*).

The ultrafast mode conversion can be ascribed to the strong NA effect that intrinsically exists in polaronic materials (*46*), i.e., the strongly coupled electron-phonon dynamics. Two theoretical simulation methods are adopted to support the above arguments, and the crucial distinction is whether the NA effect is considered or not (note S6). In both cases, the time-dependent evolutions start from the same artificial atomic structure with finite atomic displacements along the eigenvector of the *TO1@*Γ mode superposed to the IP state.

**Fig. 4.**
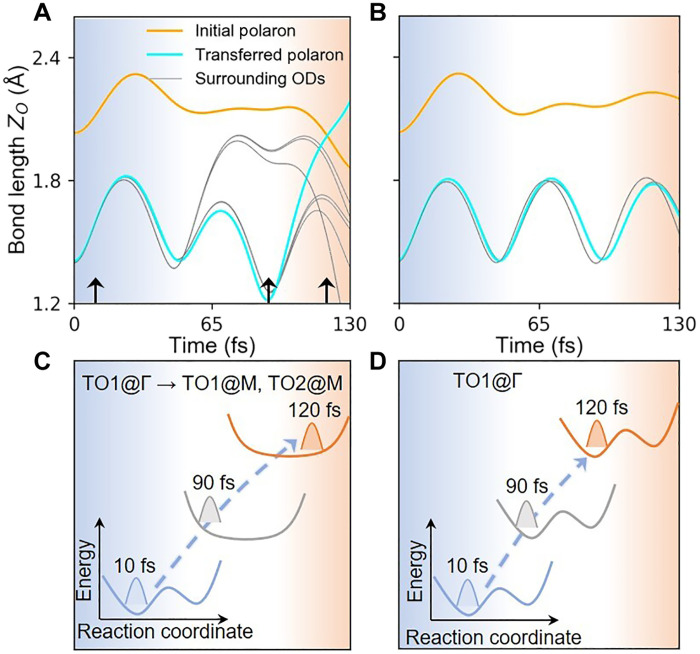
NA effect during polaron transfer. (**A** and **B**) Time evolution of *Z_O_* along the MD trajectory with (A) and without (B) NA effect. The shadow regions with different colors label the different time intervals and black arrows label the moment at 10, 90, and 120 fs. (**C** and **D**) Schematics of time-dependent PES with or without phonon scattering (for practical simulation data, see fig. S9 and note S6).

It is obvious that ultrafast polaron transport occurs only when NA effect tunes the electronic structure in real time (Fig. 4, A and B). The out-of-plane stretch of ODs before polaron transfer induces obvious oscillation of electronic bands around the Fermi surface due to strong EPI, and the nonequilibrium carrier distribution is generated along NA trajectory (e.g., at *t* = 75 fs, fig. S9C).The inhomogeneous distribution of charge density difference between the NA charge (ρ_NA_) and that obtained under adiabatic approximation (ρ_A_), i.e., Δρ = ρ_NA_ − ρ_A_, can disrupt the synchronized vibrations among ODs (fig. S9G). It leads to the strong damping of *TO1@*Γ mode, while the enhancement of *TO2@*Γ, *TO1@M*, and *TO2@M* modes emerges with intense anharmonic oscillations within ~100 fs (fig. S9A). Meanwhile, the double-well PES is modified to resemble a single-well shape with nearly diminished *E*_H_, and the system is driven to cross the TS state (Fig. 4C and note S6), which is consistent with the physical pictures obtained from the static PES upon single mode excitation (Fig. 3). In the adiabatic approximation, however, the electronic structure remains at its ground state, which would break down in describing the phonon anharmonicity at the nonequilibrium condition (fig. S9B), and thus, polaron transport is blocked by the high energy barrier of rigid double-well PES (Fig. 4D).

Thanks to the spontaneous phonon scatterings enabled by the prominent NA effect, the efficiency of laser-controlled polaron transport is determined by the photoexcited amplitude of *TO1@*Γ mode, and the phase diagram of which as functions of laser photon energy and peak amplitude is shown in Fig. 1B. The optimized photon energy (~3.3 eV) leads to nonequilibrium carrier occupations with the strongest electron-phonon couplings (note S4), and the enhancement of peak amplitude also induces the increment of phonon amplitude due to the increased nonequilibrium carrier density. Since the lifetime of coherent phonons is about tens to hundreds of picoseconds (*43*–*45*), while laser-driven polaron transfer is completed within 100 to 300 fs, the photoexcited system can be considered reaching a quasi-equilibrium steady state. Limited by the status quo that the calculation method of many physical properties in a nonequilibrium dynamic system is not yet fully established, we could roughly estimate laser-driven polaron mobility based on the Marcus theory (note S5), which is believed to be applicable only in equilibrium conditions. By considering the most dominant factor, i.e., the variation in hopping energy barrier upon photoexcitation, the qualitative comparison of polaron mobility in quasi-equilibrium systems under different laser parameters would be reasonable. It is clear that polaron mobility soars about eight orders of magnitude with *E*_0_ tuned from 0 V/Å to 0.04 V/Å at ℏω = 3.3 eV (Fig. 1C), while the effective lattice temperature in the nonequilibrium system ($\tilde{T}$, see Materials and Methods) only increases <100 K. Non-adiabatic effect would further magnify the enhancement in electrical conductivity upon photoexcitation (note S6). Polaron can hop between two pairs of ODs located at different layers along the *c* axis or in the same layer in Li_2_O_2_. The hopping direction is determined by the relative amplitude of *TO1* and *TO2* modes with nonzero wave vectors. By enhancing the laser-induced *TO1@*Γ mode, phonon-phonon scattering would be accelerated with enhanced anharmonic vibrations. The long duration of coherent phonons (*30*, *47*–*49*) and the high repetition of laser pulses all help the overall enhanced conductivity. The great tunability of electrical conductivity upon laser irradiation would stimulate the design of optoelectronics with superhigh on-off ratio and ultrafast responsibility (schematic in Fig. 1C), promising for application as photodetectors (*50*), photo-switch, and ultrafast memories.

## DISCUSSION

The significance of coherent phonon excitation can be identified by comparing it with thermal excitation, where contribution of phonons obeys the Bose-Einstein distribution with the low-energy acoustic modes being dominant, and elevating temperature (by ~100 K) only enables two orders of magnitude acceleration of polaron mobility (*12*). As for the photo-generated carriers, apart from triggering the coherent atomic vibrations, its individual contribution to polaron transfer might be marginal because of the small excitation density (fig. S5), which can be evidenced by the robust ultrafast polaron transfer even though only coherent atomic vibrations are considered (Fig. 4A). We did not directly excite polaron states into the conduction band (i.e., nonresonant excitation to polarons), and thus, the nonequilibrium polaron transfer is irrelevant to photoinduced charge de-trapping and re-trapping process, which possibly induces polaron migration, but the mobility is hard to be controlled by laser parameter. Since polaron dynamics are dominantly controlled by EPIs, the weakened electron-electron correlation (*51*–*53*) upon photoexcitation has a minor impact on its transport.

The phonon-mediated polaron transport has also been verified in a larger 4 × 2 × 1 supercell (see note S7 and fig. S10). Since the ground-state properties of polarons are negligibly affected when polaron density is reduced from 3.3 × 10^21^ cm^−3^ (one excess electron per 2 × 2 × 1 supercell) to 4 × 10^20^ cm^−3^ (one excess electron per 4 × 4 × 2 supercell), we expect that the strategy of laser-enhanced polaron transport is applicable at an experimentally attainable polaron density. The effect of laser-enhanced polaron mobility would be more pronounced when the polaron density is reduced, due to the weakened Coulomb repulsive interactions during charge transfer.

Owing to the strong EPI with band-edge carriers, excitation of *TO1* and *TO2* modes in Li_2_O_2_ contributes to polaron formation upon charge doping (*36*, *37*) and thus could facilitate polaron transport since their eigenvectors directly overlap with the polaronic lattice distortion. The patterns of lattice deformation between electron and hole polarons in Li_2_O_2_ are similar, both correlating with the variation in OD bond length along the *c* axis but in an opposite direction. Therefore, selective excitation of *TO1@*Γ modes also promotes hole polaron transfer in Li_2_O_2_ (see note S8 and fig. S11). Considering the fact that during the operation of Li-air battery, hole polarons are strongly bound to Li vacancies, complicating their transport processes (*54*), we mainly focus on the electron polarons in Li_2_O_2_ in the present work.

In combination with advanced experimental (*30*) and theoretical techniques (*36*), the electron and phonon composition of different types of polarons are identified, and thus, the laser-controlled polaron transport strategy as proposed in this work can be extended to other polaronic materials. For example, by selectively exciting the A_1g_ mode, NA effect–assisted polaron transfer is confirmed in the rutile TiO_2_ (see note S9 and figs. S12 and 13). In metal oxides serving as promising platforms for energy conversion, e.g., TiO_2_ and α-Fe_2_O_3_ (*11*, *13*), photoexciting the stretch vibration of metal-oxide bonds would induce nonequilibrium carriers with higher mobility and longer lifetime due to the accelerated polaron transport, thus improving the quantum efficiency of solar cells and the photocatalytic activities. The understanding of nonequilibrium polaron dynamics would also benefit the exploration of inherent connections between polarons and topological properties (*5*).

In conclusion, the ultrafast polaron transport dynamics driven by laser-induced coherent phonon excitation is demonstrated in a paradigmatic polaronic material, Li_2_O_2_. The polaron hopping energy barrier is lowered upon the excitation of specific vibrational modes, whose eigenvectors largely overlap with polaronic lattice deformation. Besides, the strong phonon anharmonicity originating from the NA effect is also vital in achieving ultrafast polaron transport, which provides indispensable momentum to trigger the migration of polaronic deformations. Our work displays a rational strategy for ultrafast control of the nonequilibrium quasiparticles. The laser-enhanced polaron transport might provide effective schemes to further improve the efficiency of photo-catalysis and photo-electrochemistry reactions and also innovate the design principle of optoelectronic devices such as light-sensitive detectors and photo-switch devices.

## MATERIALS AND METHODS

The ground-state properties of polaron are calculated based on DFT using SIESTA software (*55*). The Perdew-Burke-Ernzerholf (PBE) exchange-correlation functional is adopted, and the self-interaction correction is considered by the DFT + U approach. The numerical atomic orbitals with double zeta polarization are employed as the basis set and the electron-nuclear interactions are described by Troullier-Martins pseudopotentials. An auxiliary real-space grid equivalent to a plane-wave cutoff of 400 Ry is adopted and a 4 × 4 × 4 *k*-mesh is applied to sample the Brillouin zone. The space group of bulk Li_2_O_2_ is *P*6_3_*/mmc*, the primitive cell of which contains four lithium atoms and four oxygen atoms, with optimized lattice constants *a* = *b* = 3.137 Å and *c*/*a* = 2.46. The electronic structure of bulk Li_2_O_2_ is composed of O_2_^2−^ and Li^+^ ions. The coupling of two oxygen 2p orbitals leads to fully occupied σ*_p_*, π*_p_*, and $\pi_{p}^{*}$ orbitals composing the valence bands, and unoccupied $\sigma_{p}^{*}$ orbitals constituting the conduction bands near the Fermi surface. The electronic bandgap is *E_g_* = 2.0 eV with the PBE functional.

To obtain a polaronic state, one additional electron is introduced into the 2 × 2 × 1 (or the 4 × 2 × 1, 4 × 4 × 2) supercell of Li_2_O_2_. In the calculations, positive jellium background is applied to avoid the Coulomb divergence and the spin polarization is considered. One pair of oxygen dimer is extraordinarily stretched to break lattice symmetry before structural relaxation. The dependence of polaron properties, e.g., charge density distribution and atomic distortions on the Hubbard *U* of the *O*_2p_ orbital, is examined, which is negligible in both the 2 × 2 × 1 and 4 × 4 × 2 supercells (see note S3, figs. S3 and S4, and table S1). Besides, the formation energy of a single polaron is estimated to be −1.52 eV (−1.57 eV) when Hubbard *U* = 0 eV (4 eV) is adopted in the 2 × 2 × 1 (4 × 4 × 2) supercell, which is comparable with previous theoretical results (*12*). On the basis of that, we adopt the standard PBE functional and 2 × 2 × 1 supercell in MD simulations and the construction of PES.

The TDDFT-MD (time-dependent density functional theory molecular dynamics) simulations are performed using the time-dependent ab initio package (*56*, *57*) as implemented in SIESTA (see note S1 for details). During dynamic simulations, the evolving time step is set to 0.025 fs for both electrons and ions in a microcanonical ensemble. The linearly polarized laser pulses are applied to the supercell of Li_2_O_2_ with the waveform

$$E(t)=E_{0}\;\cos(\omega t)\exp\left[-\frac{{\left(t-t_{0}\right)}^{2}}{2\sigma^{2}}\right]$$ (1)

Here, the photon energy is ℏω = 2.0 eV and the half-width of Gaussian wave packet σ is 4 fs. The electric field reaches its peak amplitude *E*_0_ at time *t*_0_ = 12 fs. The ℏω (*E*_0_) are tuned from 2.0 eV to 4.5 eV (0.018 V/Å to 0.044 V/Å) to construct the phase diagram of coherent phonon excitation while the thermal damage to materials is avoided.

To describe the analogous lattice disorder as that under thermal equilibrium, an effective ionic temperature $\tilde{T}$ is defined in the nonequilibrium conditions, which comes from the transient kinetic energy of all ions (*58*, *59*)

$$\tilde{T}(t)=\frac{Mv{\left(t\right)}_{\mathrm{ions}}^{2}}{3k_{B}}$$ (2)

Here, *v*(*t*)_ions_ is the transient ionic velocity, *M* is the atomic mass, and *k*_B _is the Boltzmann constant. $\tilde{T}$ is distinct from ionic temperature in thermal equilibrium *T*, but both of them are used to characterize the heating effects induced by lasers or temperature.

## Supplementary Materials

### This PDF file includes:

Supplementary notes S1 to S9 figs. S1 to S13 Table S1 References
