# Supplementary material for: Giant acceleration of polaron transport by ultrafast laser-induced coherent phonons
Source: Sci Adv. 2023 Aug 16;9(33):eadg3833. doi: 10.1126/sciadv.adg3833 (PMC10431702; doi:10.1126/sciadv.adg3833)
Supplement: Supplementary file 1 — Supplementary notes S1 to S9 figs. S1 to S13 Table S1 References [file sciadv.adg3833_sm.pdf]

Science Advances
AAAS

Supplementary Materials for

Giant acceleration of polaron transport by ultrafast laser-induced coherent phonons

Hui-Min Wang *et al.*

Corresponding author: Cui Zhang, cuizhang@iphy.ac.cn; Meng-Xue Guan, mxguan@bit.edu.cn; Sheng Meng, smeng@iphy.ac.cn

Sci. Adv. **9**, eadg3833 (2023)
DOI: 10.1126/sciadv.adg3833

.

This PDF file includes:

Supplementary Notes S1 to S9
Figs. S1 to S13
Table S1
References

Note S1. Nonadiabatic dynamics with time-dependent density functional theory (TDDFT)

During the real-time evolutions, the nonadiabatic couplings (i.e., the correlated electron-phonon dynamics beyond the Born-Oppenheimer approximation) are governed by the Ehrenfest theorem(*63*).The electronic density is propagated in real-time through numerical integration of TDKS equations, meanwhile the ionic motion is calculated according to the law of classical mechanics on a potential energy surface given by the expectation value of the electronic Hamiltonian $\widehat{H}_{el}$.

$$\widehat{H}_{el}(\boldsymbol{r},\boldsymbol{R}(t)) = \sum_{i} \frac{\hat{p}_i^2}{2m} + V(\boldsymbol{r},\boldsymbol{R},t) + U_{ext}(\boldsymbol{r},\boldsymbol{R},t), \quad \text{(S1)}$$

$$V(\boldsymbol{r},\boldsymbol{R},t) = \sum\nolimits_{i<j} \frac{e^2}{|\boldsymbol{r}_i - \boldsymbol{r}_j|} - \sum\nolimits_{i,\alpha} \frac{eZ_\alpha}{|\boldsymbol{r}_i - \boldsymbol{R}_\alpha|} + \sum\nolimits_{\alpha,\beta} \frac{Z_\alpha Z_\beta}{|\boldsymbol{R}_\alpha - \boldsymbol{R}_\beta|}. \quad \text{(S2)}$$

Here, $\sum_{\boldsymbol{i}} \frac{\hat{p}_i^2}{2m}$ is the electronic kinetic energy, and $U_{ext}(\boldsymbol{r},\boldsymbol{R},t)$ is external potential, where, *m* (*e*) is the mass (charge) of electron and Z_α is the charge of αth nuclear. $\boldsymbol{R} = (\boldsymbol{R_1},\boldsymbol{R_2},\dots,\boldsymbol{R_N})$ and $\boldsymbol{r} = (\boldsymbol{r_1},\boldsymbol{r_2},\dots,\boldsymbol{r_N})$ are the collective coordinates of nuclear position $\boldsymbol{R_\alpha}$ and electronic position $\boldsymbol{r_i}$, respectively.

The forces on the nuclei are

$$F_{\boldsymbol{\alpha}} = -\sum_{m} \langle \varphi_m | \nabla_\alpha \widehat{H}_{el} | \varphi_m \rangle, \quad \text{(S3)}$$

in which φ_m describes the time-dependent electronic wavefunction,

$$i\hbar \frac{\partial}{\partial t} \varphi_m(\boldsymbol{r},\boldsymbol{R},t) = \widehat{H}_{el}\big(\boldsymbol{r},\boldsymbol{R}(t)\big)\varphi_m(\boldsymbol{r},\boldsymbol{R},t). \quad \text{(S4)}$$

According to the Runge-Gross theorem (*64*), Eq. S4 can be reduced into the time-dependent Kohn-Sham (TDKS) equation:

$$i\hbar \frac{\partial}{\partial t} \psi_m(\boldsymbol{r},t) = \widehat{H}_{KS}\big(\boldsymbol{r},\boldsymbol{R}(t)\big)\psi_m(\boldsymbol{r},t), \quad \text{(S5)}$$

where $\psi_m(\boldsymbol{r},t)$ are the TDKS orbitals and

$$\widehat{H}_{KS}\big(\boldsymbol{r},\boldsymbol{R}(t)\big) = \sum_{i} \frac{\hat{p}_i^2}{2m} + V_{KS}(\boldsymbol{r},t) + U_{ext}(\boldsymbol{r},\boldsymbol{R},t), \quad \text{(S6)}$$

$$V_{KS}(\boldsymbol{r},t) = \sum V_\alpha^{KB} + \sum V_\alpha^{local}(\boldsymbol{r}) + \sum V^{xc}(\boldsymbol{r},t) + \sum V^H(\boldsymbol{r},t). \quad \text{(S7)}$$

Here, V_α^{KB} and $V_\alpha^{local}(\boldsymbol{r})$ are the Kleinman-Bylander and local parts of the pseudopotential of atom α, while $V^{xc}(\boldsymbol{r},t)$ and $V^H(\boldsymbol{r},t)$ are the exchange-correlation (XC) and Hartree potentials, respectively.

Light-matter interactions are considered by adding external potential $U_{ext}(\boldsymbol{r}, \boldsymbol{R}, t)$ into the electronic Hamiltonians. To deal with periodic systems, velocity gauge is used, where the relation between vector and scalar potential of the electric field $\boldsymbol{E}(t)$ is $\boldsymbol{A}(t) = -c\int_0^t \boldsymbol{E}(t')dt'$ and $\Phi = 0$. Time evolution of electronic wavefunctions is computed by propagating the TDKS equations (Eq. S5) in atomic units (a.u.),

$$i\frac{\partial}{\partial t}\psi_m(\boldsymbol{r}, t) = [\frac{1}{2m}\left(\boldsymbol{p} - \frac{e}{c}\boldsymbol{A}\right)^2 + V_{KS}(\boldsymbol{r}, t)]\psi_m(\boldsymbol{r}, t). \quad \text{(S8)}$$

Ionic forces along the classical trajectory evaluated through the Ehrenfest theorem is

$$-F_\alpha = \int d\boldsymbol{r}\ \psi_m^*(\boldsymbol{r}, t)\nabla_\alpha \hat{\mathrm{H}}_{KS}(\mathbf{r}, \mathbf{R}(\mathrm{t}))\psi_m(\boldsymbol{r}, t). \quad \text{(S9)}$$

The TDKS equations of electrons (Eq. S8) and the Newtonian motion of ions (Eq. S9) are solved simultaneously, enabling the real-time tracking of coupled electron-nuclear dynamics under non-equilibrium conditions.

Compared to other software based on the Ehrenfest theorem (*65, 66*), the home-made software (TDAP) has superior computational efficiency due to the adopted numerical atomic orbital basis, which enabled the time-dependent simulations of electron-nuclear dynamics in complex systems (up to 1000 atoms) on the order of picoseconds with attosecond and angstrom resolutions.

Note S2. Ground-state properties of polarons in Li_2O_2 obtained in different size of simulation cell

Figure S1(A-D) shows the charge density distribution of excess electrons in 2×2×1 and 4×4×2 supercells, which are mainly localized at one of the O-O dimers (OD), i.e., the center of polaron, leading to the bond breaking along the *c*-axis in both two cases. O_2^{3-} forms at the location of electron polaron, while the valence states of Li^+ ions are nearly unchanged. Meanwhile, a small fraction of excess electrons is distributed around the surroundings of OD, resulting in slightly contracted bonds. The characteristic lattice distortion as well as the charge distribution fit well with previous theoretical works (*12,26,36*). The out-of-plane elongation of an OD breaks degeneracy of σ_p^* orbitals with an σ_p^* orbital lowered in energy, where an excess electron occupies a spin channel, leaving the other spin channel to be empty, forming the occupied and unoccupied polaronic bands as shown in Fig. S1F and a local magnetic moment of $1\mathrm{u}_B$. The generation of non-equilibrium carriers due to NA effects is feasible for both two spin channels. To achieve polaronic charge transfer, electrons are excited from one to another σ_p^* orbitals in the same spin channel since the spin-orbital couplings in Li_2O_2 are too weak to be considered during the dynamic evolution within sub-picoseconds (Note S6 and Fig. S9).

The quantitative comparison of the ground-state properties of polaron can be obtained by analyzing the bond lengths of four nearby ODs and one-dimensional polaron charge distributions in small and large supercells. It is clear that the bond length differences are nearly negligible (<1.4%, Fig. S1E) and the profile of polaron densities along *b*-axis is identical (Fig. S2A). Note that although the smaller 2×2×1 supercell fails to perfectly reproduce charge density distributions far away from the polaron center along the *c*-axis, the properties of polaron would not be changed due to its relatively small size (Fig. S2C). Besides, a general analysis of atomic distortions (including the displacement of oxygen and lithium atoms around the polaron) also reveals a negligible difference (Fig. S2(B, D)).

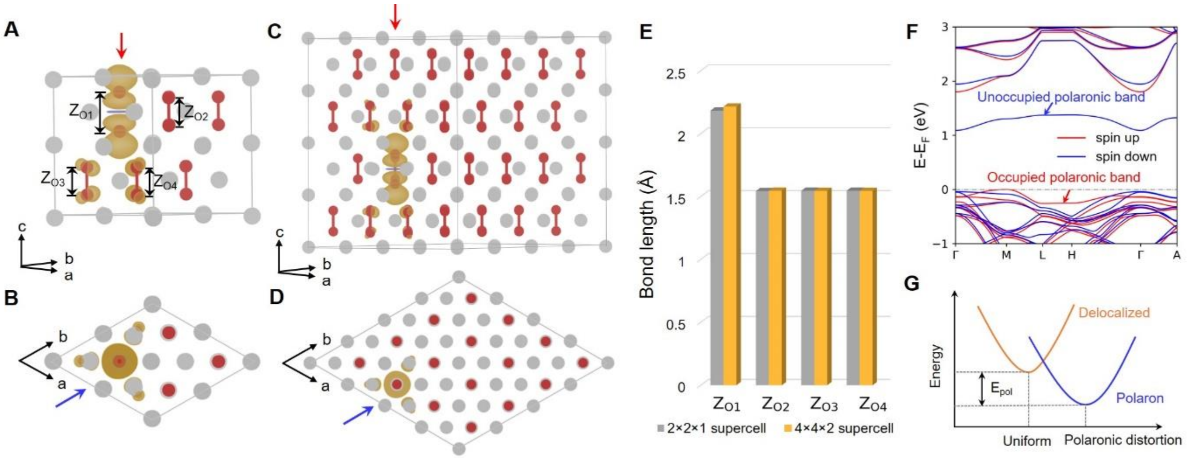

Fig. S1. Ground-state properties of polarons in Li_2O_2. (**A-B**) Side and top views of optimized atomic structure and charge density distribution of polaron in the $2\times2\times1$ supercell. The red and gray balls represent oxygen and lithium atoms, respectively. The bond lengths of four O-O dimers nearest to the polaron are labeled as Z_{Oi} (i=1,2,3,4). The yellow area shows the charge density with isosurface of 7×10^{-3} e/Å^3. (**C-D**) are analogous to (**A-B**) but for the $4\times4\times2$ supercell. (**E**) Comparison of Z_{Oi} obtained in the two simulation cells. (**F**) Spin polarized band structure. The spin up (down) channel is plotted by red (blue) line. (**G**) Energy landscape for polaron formation. The energy difference between the polaron and the delocalized electronic state with undistorted lattice structure is defined as the polaron formation energy (E_{pol}).

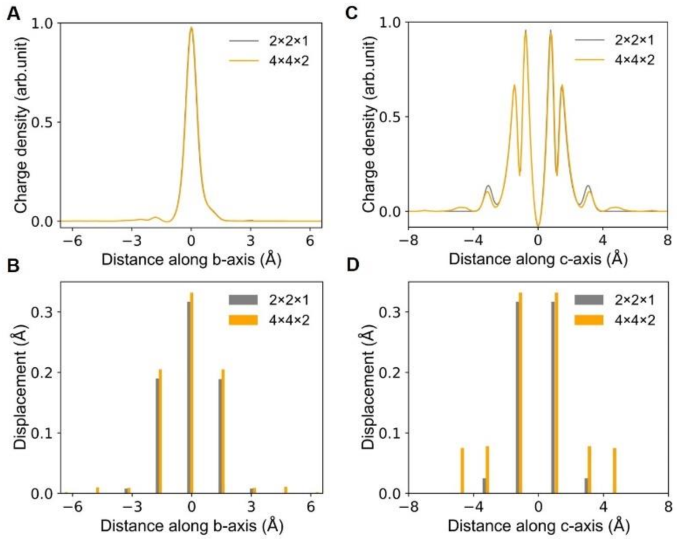

Fig. S2. Quantitative comparison of polaron properties obtained in different size of simulation cells. (**A**) One-dimensional profile of the polaron density in the two supercells along the crystalline *b*-axis, i.e., blue arrows shown in Fig. S1(**B**, **D**). The periodic repetition of the lattice is considered and the position of polaron center is set to be zero for clarity. (**B**) Atomic displacements along the same spatial positions as that in (**A**). (**C-D**) are analogous to (**A-B**) but along the line indicated by the red arrows in Fig. S1(**A**, **C**).

Note S3. Ground-state properties of polarons in Li_2O_2 obtained by different *U* value

To examine the effects of self-interaction correction (SIC) in the present work, the polaron density and atomic distortions in both the 2×2×1 and 4×4×2 supercells are calculated based on both PBE and PBE+U approaches, as shown in Fig. S3-S4 and Table S1. It is clear that the ground-state properties of polaron with different Hubbard *U* are nearly identical. For example, when $U = 4$ eV is applied to the O_{2p} orbital in the 4×4×2 supercell, the most important correction only accounts for ~ 1% change in Z_{O1}, compared with our previous results (performed with standard PBE calculation in a 2×2×1 supercell).

We have further compared our results with previous theoretical works adopting different SIC methodologies, e.g., HSE (*12*), *ab initio* SIC (*36*), and larger Hubbard *U* (*26*). Summarizing all these available data, it shows that among various methods: i) the relative differences in the polaronic distortions (i.e., the bond length of the OD where polaron locates Z_{O1}) are < 3.5 % (Table S1). ii) The one-dimensional profile of polaron density matches well with that obtained by the advanced *ab initio* formalism for polarons (*36*) (Fig. S4(C and D)).

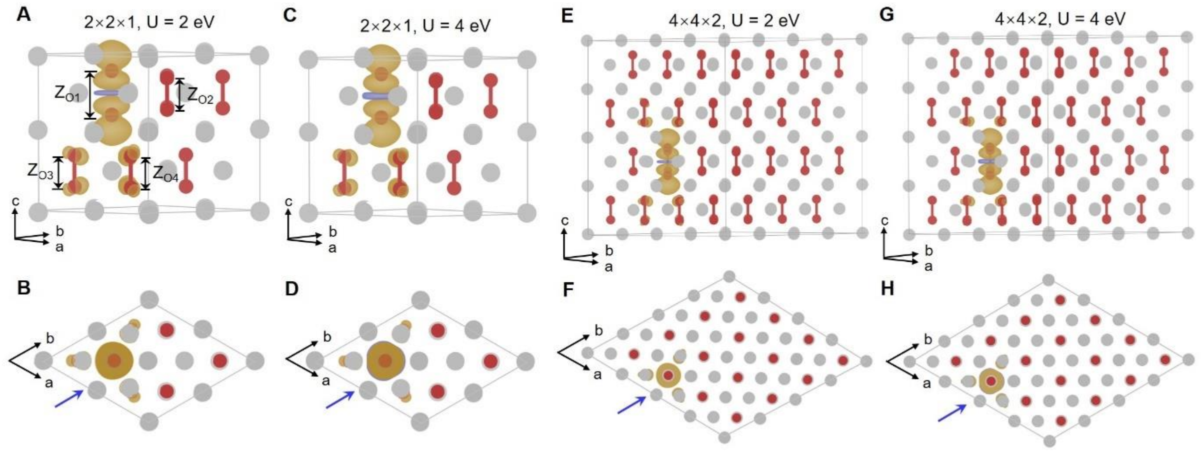

Fig. S3. Ground-state properties of polarons obtained by different *U* value. (**A-B**) Side and top views of optimized atomic structure and charge density distribution of polaron obtained in the 2×2×1 supercell with $U = 2$ eV. The yellow area shows the charge density with isosurface of 7×10^{-3} e/Å^3. (**C-D**), (**E-F**), (**G-H**) are analogous to (**A-B**) but for different simulation variables.

Bond-length (Å)	2×2×1 U = 0 eV	2×2×1 U = 2 eV	2×2×1 U = 4 eV	4×4×2 U = 0 eV	4×4×2 U = 2 eV	4×4×2 U = 4 eV	4×3×2 HSE (*12*)	3×3×3 ab initio SIC (*36*)	4×4×2 DFT+U (6 eV) (*26*)
Z_{O1}	2.183	2.174	2.174	2.214	2.206	2.206	2.20	2.25	2.18
Z_{O2}	1.543	1.550	1.556	1.546	1.553	1.560	/	/	/
Z_{O3}	1.546	1.550	1.556	1.545	1.551	1.557	/	/	/
Z_{O4}	1.546	1.550	1.556	1.545	1.551	1.557	/	/	/

Table. S1. Comparison of O-O dimer bond-length (Z_{Oi}) with different simulation set-ups.

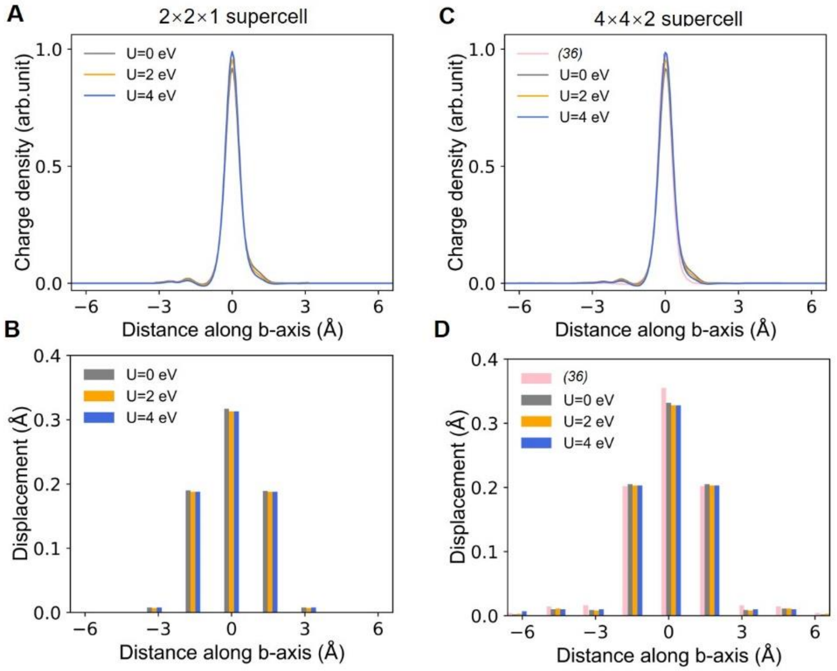

Fig. S4. Quantitative comparison of polaron properties obtained by different *U* value. (**A**) One-dimensional profile of the polaron density along the line indicated by the blue arrows in Fig. S3(**B**, **D**) with 2×2×1 simulation cell for different Hubbard *U*. The periodic lattice repetition is considered and the position of polaron center is set to be zero for clarity. (**B**) The atomic displacements along the same direction used in (**A**). (**C-D**) are analogous to (**A-B**) but for the 4×4×2 supercell. Here, the pink bar (lines) shows the results obtained by *ab initio* polaron equation (*36*).

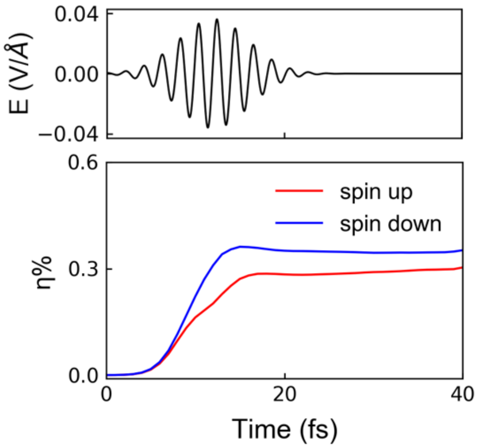

Fig. S5. Laser profile and amount of photoexcited electrons (η). The applied electric field along the crystalline b-axis reaches its peak amplitude $E_0 = 0.036$ V/Å at $t_0 = 12$ fs with the pulse width $\sigma = 4$ fs and photon energy $\hbar\omega = 2.0$ eV. η is defined as the proportion of valence electrons pumped to the unoccupied conduction bands.

Note S4. Electron-phonon interaction (EPI) and atomic forces

Photodoping induced atomic forces $\boldsymbol{F}_\alpha$ triggers coherent phonons, and thus its selective excitation pathway can be designed based on electron-phonon interaction analysis.

$$\boldsymbol{F}_\alpha = -\frac{1}{N}\sum_{i,\mathbf{k}} \Delta f_{ik}\, g_{ii}^{\boldsymbol{k}}, \qquad \text{(S10)}$$

$$g_{ii}^{\boldsymbol{k}} = \langle i\mathbf{k}|\nabla_{\tau_\alpha}\widehat{H}|i\mathbf{k}\rangle. \qquad \text{(S11)}$$

Here, i and $\boldsymbol{k}$ are the band index and the wave-vector in the momentum space, τ_α is the atomic displacement of atom α and $\widehat{H}$ is the Hamiltonian. $\boldsymbol{F}_\alpha$ on atom α depends on EPI matrix element $g_{ii}^{\boldsymbol{k}}$ and the non-equilibrium carrier population Δf_{ik} as compared with the ground-state.

In Li_2O_2, upon laser irradiation with photon-energy $h\omega = 2.0$ eV, the non-equilibrium electrons and holes locate around the *Γ* point of the lowest conduction band (LCB) and the highest valence band (HVB), respectively (Fig. S6E). The corresponding EPI matrix elements for four zone-center *TO* modes are shown in Fig. S6C. The *TO1@Γ* mode owes the largest g_{ii}, while that of other *TO* modes are nearly zero, which means that upon the resonant transition of carriers ($h\omega =$ 2.0 eV), the *TO1@Γ* mode will be predominantly excited.

Due to the band dispersion of Li_2O_2 (Fig. S6A), changing photon energy from 2.0 eV to 4.0 eV corresponds to carrier transition between the HVB and LCB but at different $\boldsymbol{k}$ points. The $\boldsymbol{k}$-resolved $g_{ii}^{\boldsymbol{k}}$ for *TO1@Γ* mode shows that carriers occupy at L, H two points have the strongest EPI matrix element (Fig. S6D), corresponding to carrier transition under $h\omega =$ 3.3 ~ 4.0 eV (Fig S6 (F and G)). In combined with the non-equilibrium carrier density, which also depends on the photon energy (Fig. S6H), the most efficient phonon excitation for *TO1@Γ* mode is at $h\omega \approx 3.3$ eV.

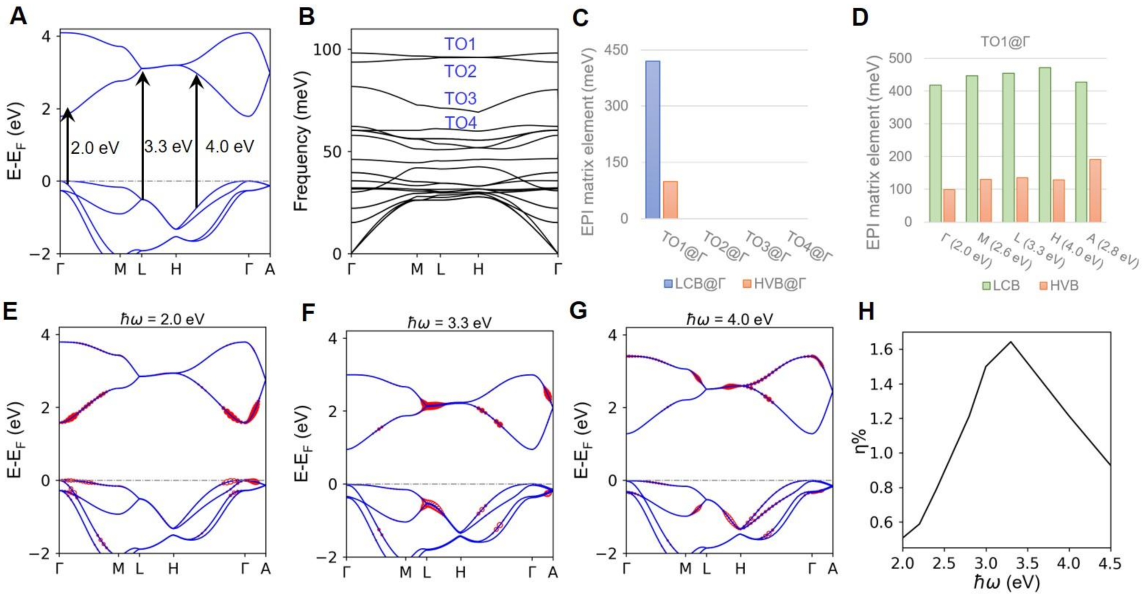

Fig. S6. Electron-phonon interactions (EPI) in Li_2O_2. (**A-B**) The electronic band structure and phonon spectrum of bulk Li_2O_2, respectively. The carrier transition pathways under different photon energy and the top four optical modes are labeled. (**C**) EPI matrix elements of four zone-center *TO* modes with electron (holes) occupying at the *Γ* point of LCB (HVB). (**D**) EPI matrix element of *TO1@Γ* mode with carriers occupying the LCB and HVB but at different $\boldsymbol{k}$ point. The corresponding photon energies are labeled. (**E-G**) The non-equilibrium carrier occupations at the end of laser filed (t = 25 fs) with $h\omega = 2.0, 3.3$ and 4.0 eV, respectively. (**H**) The non-equilibrium carriers amount as a function of photon energy with laser peak amplitude set as 0.036 V/Å.

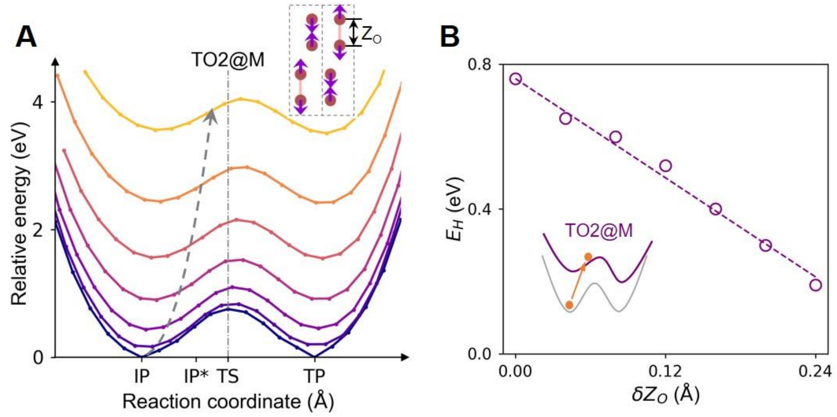

Fig. S7. The role of *TO2@M* mode in polaron transfer. (**A**) Modulated PES due to phonon excitation of *TO2@M* mode with different amplitudes of δZ_O .The gray dashed lines show the modified position of the initial polaron (i.e., IP*) along the hopping trajectory with increased phonon amplitude. (**B**) Dependence of E_H on the δZ_O, where the circles are calculation data and dashed line are their linear fit. The insets schematically depict the PES with and without phonon excitations.

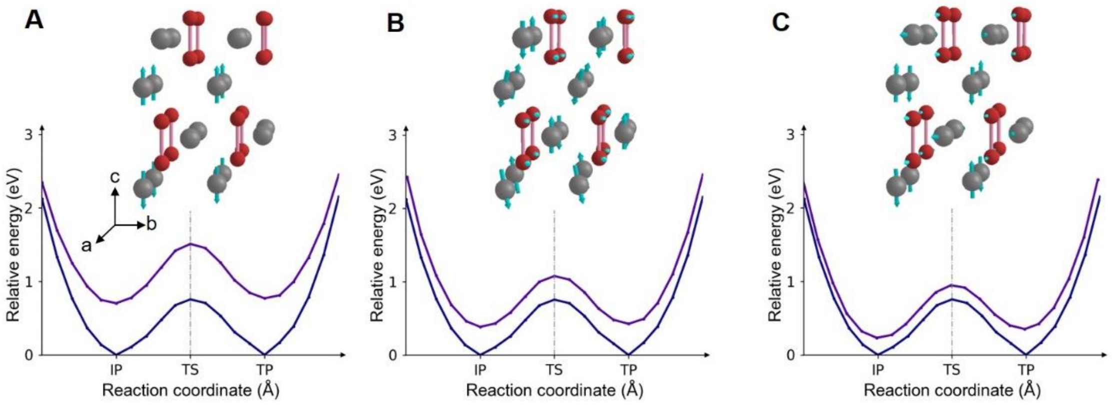

Fig. S8. Potential effect of the optical modes that not involved in polaron transfer. (**A-C**) The vibrational eigenvectors of phonon modes are shown, which are related with the motions of lithium atoms (gray balls) and shear motions of oxygen atoms (red balls). The excitation of these modes only induces inconsiderable change of the PES (purple lines), as compared with the thermal equilibrium condition (navy blue lines).

Note S5. The carrier mobility via polaron hopping

The polaron mobility (μ) can be estimated via the Einstein relation:

$$\mu = \frac{eD}{k_B T}, \qquad \text{(S12)}$$

where k_B is the Boltzmann constant, D is the diffusion coefficient, T is the thermal equilibrium temperature.

The diffusion coefficient of polarons is estimated via:

$$D = NR^2 \nu \, exp(\frac{-E_H}{k_B T}). \qquad \text{(S13)}$$

Here, N is the number of neighboring hopping sites (for Li_2O_2, $N = 6$), R is the distance between the hopping sites, ν is the vibrational frequency of atoms (ν ~100 meV for *TO1* mode), and $E_H^0 = 0.66$ eV for E_H in equilibrium condition. Polaron mobility under thermal equilibrium at $T = 300$ K is $\mu_0 \sim 5 \times 10^{-11}$ $\mathrm{cm^2/(v \cdot s)}$.

The linearly dependence of hoping energy barrier (E_H) on the amplitude of photoexcited *TO1* and *TO2* modes (δZ_O) (Fig. 3D and Fig. S7B) can be summarized as:

$$E_H = E_H^0 + k_i * \delta Z_O, \qquad \text{(S14)}$$

where k_i is the slope and i labels the phonon branch, $k_1 = -1.91$ eV/Å for *TO1@Γ* mode, $k_2 = -2.45$ eV/Å for *TO1@M* mode, $k_3 = -2.28$ eV/Å for *TO2@M* mode. By combining the formula S12-S14, we can estimate the non-equilibrium polaron hopping mobility, which exponentially increases with the photoexcited phonon amplitude, as shown in Fig. 1C.

Note S6. NA effect induced phonon anharmonicity

To explicitly show the importance of NA effect, theoretical simulations based on TDDFT-MD including NA effect and *ab initio* molecular dynamics with adiabatic Born-Oppenheimer (BO) approximation were performed. Following the coherent atomic vibrations along *TO1@Γ* mode (i.e., the synchronized out-of-plane stretch of ODs), electronic bands around the Fermi surface are dramatically modulated due to the strong EPI. The band gap can be transiently closed and thus the non-equilibrium carrier occupations are generated along the NA trajectory (e.g., t = 75 fs, see Fig. S9C), which in turn modulates the coherent atomic vibrations via the inhomogeneous atomic forces (Fig. S9G) and eventually induces the scattering of phonons within sub-picoseconds (Fig. S9A). In the BO approximation, however, the electronic structure assumes the ground-state (GS) along the MD trajectory, which would break down in describing the strong phonon-phonon scatterings (Fig. S9B). Therefore, the non-equilibrium electronic configuration along the NA trajectory would in-turn affect the atomic motions, leading to the NAMD trajectory gradually distinct from the BOMD trajectory.

The significance of phonon anharmonicity originating from NA effects to polaron transport is also shown by the time dependent PES (Fig. S9(D and E)). To establish the dynamic PES, we have selected atomic structures from the NAMD and BOMD trajectories with a time interval of 10 fs, and then superimpose the transient atomic distortions onto the atomic configurations interpolated along polaron transfer pathway at thermal-equilibrium. Therefore, the comprehensive influences of multiple modes excitation on polaron transfer can be revealed by the modified shape of transient PES, which is dynamically changed due to the evolution of phonon subsystem.

The metallic band structure along the NAMD trajectory forms when the *TO1@Γ* mode reaches the peak amplitude (t = 25, 70 fs) or the scattered *TO2@M* mode shows strong anharmonicity (t = 75 ~125 fs), due to the intense out-of-plane stretch of ODs, leading to obvious lower energy of σ_p^* orbitals. The transient excitation of valence electrons due to NA effects would improve electrical conductivity by increasing conductive electron density (the ratio of non-equilibrium carriers is ~0.11%), but has little impact on the polaron mobility, which is determined by phonon excitation rather than electron-electron scatterings*(2)*. NA effects are enhanced with increasing phonon amplitude due to more violent oscillation of σ_p^* orbitals. Therefore, apart from the increase in polaron mobility, the enhancement in electrical conductivity upon photoexcitation would be even more obvious by considering the NA effects.

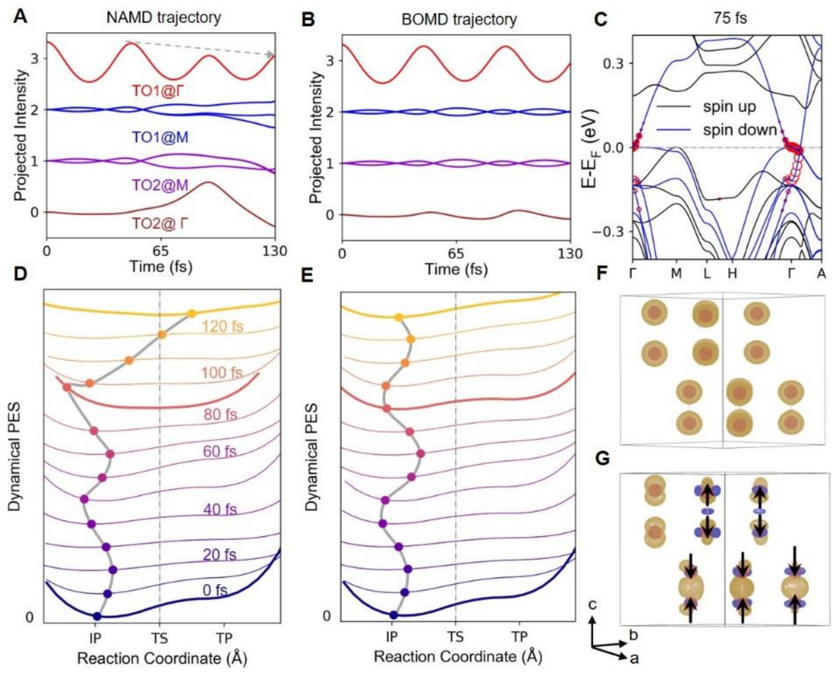

Fig. S9. NA effects during polaron transfer. (**A-B**) Projection weight of atomic displacement onto the vibrational eigenvectors along the NAMD trajectory and BOMD trajectory, respectively. The gray dashed line in (**A**) is guide for the strong damping of *TO1@Γ* mode with initial atomic displacements $\delta Z_O = 0.16$ Å. (**C**) NA effects induced non-equilibrium carrier population. The spin-polarized band structure is calculated with atomic structure extracted from the NAMD trajectory at $t = 75$ fs. The filled (hollow) circles represent the non-equilibrium electrons (holes). (**D-E**) The dynamical PES along NAMD and BOMD trajectory, respectively. The time-dependent oscillation of system along PES is labeled by color dots and linked by gray lines. The energy of each transient PES is normalized by the local minimum. (**F**) Real-space distribution of charge density at t = 75 fs along the NAMD trajectory with isosurface of $0.7\ \mathrm{e/Å^3}$. (**G**) Real-space distribution of charge density difference $\Delta\rho = \rho_{NA} - \rho_A$ at t = 75 fs, where ρ_{NA} is the NA charge and ρ_A is that obtained under adiabatic approximation. The orange and blue areas show the positive and negative $\Delta\rho$ with isosurface $1.3 \times 10^{-3}\ \mathrm{e/Å^3}$. The direction and length of black arrows indicate the direction and magnitude of corresponding atomic forces ($\Delta F = F_{NA} - F_A$).

Note S7. Phonon-driven polaron transfer in the 4×2×1 supercell

The phonon-driven polaron transfer in the non-equilibrium regime has also been directly verified with the affordable 4×2×1 supercell which contains 64 atoms, as shown in Fig. S10. Upon excitation of *TO1@Γ* mode, the crossing of Z_O between the initial and transferred polaron indicates that the transition state along polaron transfer pathway is reached, which is well consistent with the results obtained in the 2×2×1 supercell (Fig. 4A).

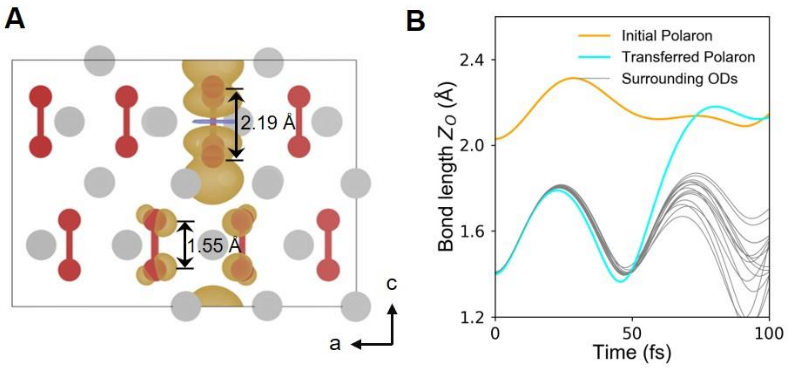

Fig. S10. Static properties and transfer dynamics of polarons in 4×2×1 supercell. (**A**) Optimized atomic structure and charge density distribution of the polaron. The red and gray balls represent oxygen and lithium atoms, respectively. The yellow area shows the charge density with isosurface of 7×10^{-3} e/Å^3. (**B**) The time evolution of Z_O along the TDDFT-MD trajectories. The initial state of MD trajectories is built up via stretching atomic displacement along the *TO1@Γ* mode (same as Fig. 4A).

Note S8. Phonon-driven hole polaron transfer in Li_2O_2

The hole polarons are simulated by removing an electron from the 4×4×2 supercell of Li_2O_2 (containing 256 atoms) and adding Hubbard *U* correlation to the O 2p orbital with *U* = 7 eV. Hole polaron localizes at one of the ODs, inducing its contraction along the out-of-plane direction by 0.17 Å, consistent with previous theoretical studies(*26*). The patterns of lattice deformation between electron and hole polarons in Li_2O_2 are similar, both correlating with the variation in OD bond length along *c*-axis but in an opposite direction. Therefore, similar vibrational modes (*TO1* and *TO2* modes) contribute to their transport. This speculation is confirmed by the change of phonon-modulated PES along a hole polaron transfer pathway (see Fig. S11B) and the real-time TDDFT-MD simulations which start from atomic structure with stretched *TO1@Γ* mode onto the initial hole polaron state (Fig. S11C). It is shown that hole transfer occurs at *t* = 65 fs.

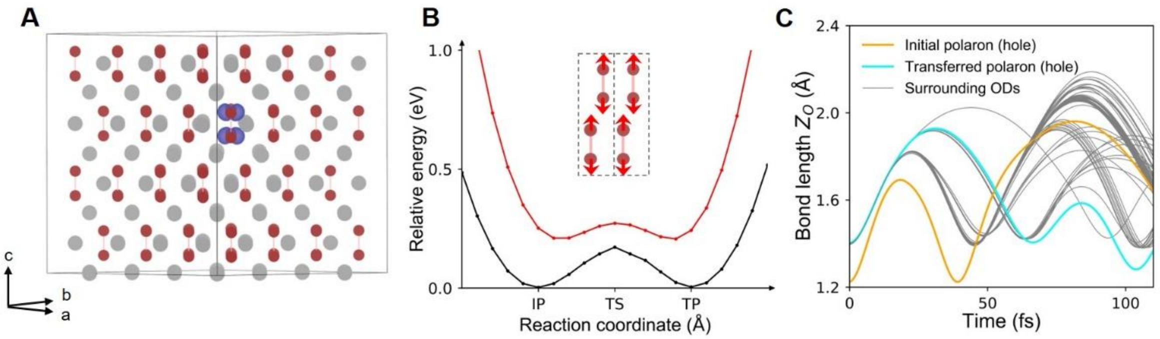

Fig. S11 Phonon-driven hole polaron transfer in Li_2O_2. (**A**) The optimized atomic structure and charge density distribution of the hole polaron with isosurface of 7×10^{-2} e/Å^3. (**B**) Comparison of equilibrium PES (black line) and that modulated upon the excitation of *TO1@Γ* mode (red line) with amplitude $\delta Z_O = 0.16$ Å. The hopping energy barrier is reduced due to excitation of *TO1@Γ* mode. (**C**) Time evolution of Z_O, starting from atomic structure with stretched *TO1@Γ* mode ($\delta Z_O = 0.16$ Å) onto the initial hole polaron state.

Note S9. Laser-controlled polaron transfer in rutile TiO_2

The electron polaron in rutile TiO_2 (r-TiO_2) is simulated by adding an excess electron into the 2×2×3 supercell (containing 72 atoms) and adopting Hubbard *U* correction for Ti *3d* orbital with *U* = 4.2 eV. The stably formed polaron (defined as the IP) shows as an excess electron localized at one of Ti atoms, and the bond length between Ti and O atoms in this octahedron is stretched, i.e., four Ti-O1 (equatorial oxygens) bonds elongated by 0.1 Å and two Ti-O2 (axial oxygens) bonds elongated by 0.03 Å (Fig. S12A). Based on the main feature of polaronic lattice distortions, the A_{1g} mode is identified as the key mode contributing to polaron transfer, which characterizes the simultaneous stretch of Ti-O1 bonds and contraction of Ti-O2 bonds in all octahedrons (Fig. S12B). The critical role of exciting A_{1g} mode in promoting polaron transfer is confirmed by the variation in PES with reduced hopping energy barrier (Fig. S12C). The BOMD and NAMD trajectories are evolved starting from the same atomic structure with finite atomic displacements along the eigenvector of A_{1g} mode superposed to the IP state (see Fig. S12(D-F)). Strong indication of polaron transfer is shown at *t* = 55 fs along the NAMD trajectory, where the transition state is approaching, i.e., localized electrons are nearly equally distributed at two Ti atoms.

The selective photoexcitation of A_{1g} mode is achieved due to the strongest electron-phonon couplings when electrons (holes) are distributed at the band-edge (Fig. S13(B and C)). The amplitude of excited A_{1g} mode can be laser-controlled by tuning the amounts and distribution of non-equilibrium carriers under different laser-parameters (Fig. S13D).

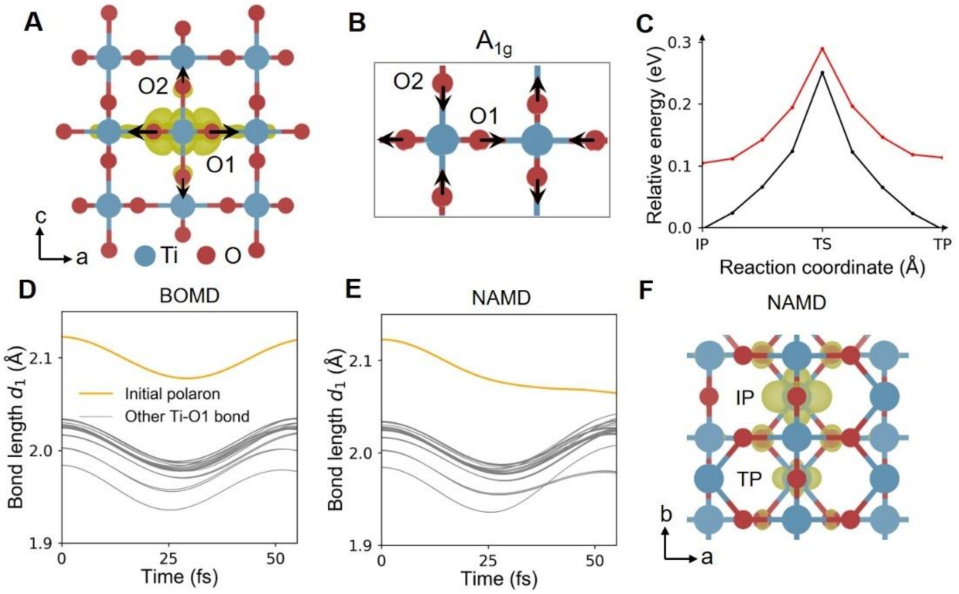

Fig. S12 Phonon-driven polaron transfer in rutile TiO_2. (**A**) Optimized atomic structure and charge density distribution of electron polaron. The red and blue balls represent oxygen and titanium atoms, respectively. The equatorial (axial) oxygen atoms in Ti-O octahedron are labeled

as O1(O2), with the bond length of Ti-O1(O2) defined as d_1 (d_2). The yellow area shows the charge density with isosurface of 3×10^{-2} e/Å^3. The black arrows are guides for the direction of polaronic deformations. (**B**) The eigenvector of A_{1g} mode, largely overlapping with the pattern of polaronic deformations. (**C**) Comparison of thermal equilibrium PES for polaron transfer (black line) and that modulated upon the excitation of A_{1g} mode (red line). Here, the variation in d_1 (δd_1) used to define the amplitude of A_{1g} mode is 0.1 Å. (**D-E**) Time evolution of d_1 along BOMD (**D**) and NAMD (**E**) trajectories, starting from the same lattice structure with finite atomic displacements along the eigenvector of A_{1g} mode ($\delta d_1 = 0.04$ Å) superposed to the IP state. (**F**) Charge density distribution of polaron at t = 55 fs along NAMD trajectory with isosurface of 3×10^{-2} e/Å^3, indicating it is close to the transition state (TS).

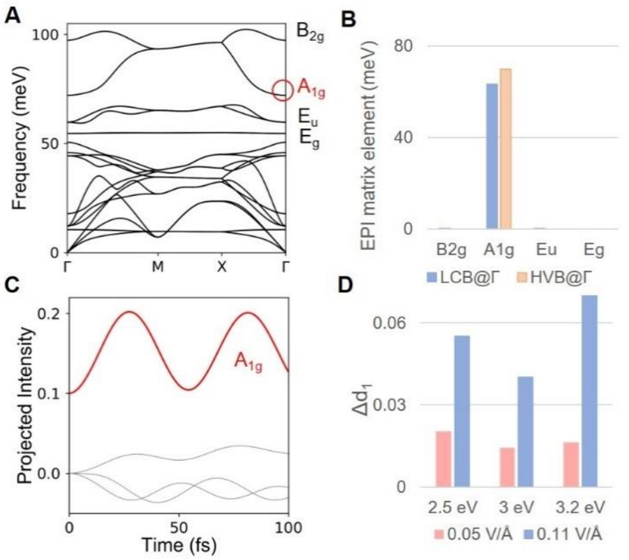

Fig. S13 Laser-controlled selective excitation of A_{1g} mode in the rutile TiO_2. (**A**) Phonon spectrum of bulk TiO_2. The top four optical modes at the *Γ* point are labeled with irreducible representations. (**B**) EPI matrix elements of the four modes with electrons and holes occupying at *Γ* point of the lowest conduction band (LCB) and highest valence band (HVB). (**C**) Projected intensity of atomic displacements onto vibrational eigenmodes upon photoexcitation. The applied electric field along the crystalline *b*-axis reaches its peak amplitude $E_0 = 0.077$ V/Å at $t_0 = 12$ fs with the pulse width $\sigma = 4$ fs and photon energy $\hbar\omega = 2.5$ eV. Here, $\delta d_1 = 0.04$ Å when A_{1g} mode reaches the peak amplitude. (**D**) Phonon amplitude of A_{1g} mode as a function of photon energy $\hbar\omega$ and laser peak amplitude E_0.

REFERENCES AND NOTES

1. C. Franchini, M. Reticcioli, M. Setvin, U. Diebold, Polarons in materials. *Nat. Rev. Mater.* **6**, 560–586 (2021).

2. D. Emin, *Polarons* (Cambridge Univ. Press, 2013).

3. A. S. Alexandrov, *Polarons in Advanced Materials* (Springer, 2007).

4. L. P. Rene de Cotret, M. R. Otto, J.-H. Pöhls, Z. Luo, M. G. Kanatzidis, B. J. Siwick, Direct visualization of polaron formation in the thermoelectric SnSe. *PNAS* **119**, e2113967119 (2022).

5. P. Suo, H. Zhang, S. Yan, W. Zhang, J. Fu, X. Lin, S. Hao, Z. Jin, Y. Zhang, C. Zhang, F. Miao, S.-J. Liang, G. Ma, Observation of negative terahertz photoconductivity in large area type-II Dirac semimetal $PtTe_2$. *Phys. Rev. Lett.* **126**, 227402 (2021).

6. I. N. Hulea, S. Fratini, H. Xie, C. L. Mulder, N. N. Iossad, G. Rastelli, S. Ciuchi, A. F. Morpurgo, Tunable Fröhlich polarons in organic single-crystal transistors. *Nat. Mater.* **5**, 982–986 (2006).

7. W. Jin, H. H. Kim, Z. Ye, G. Ye, L. Rojas, X. Luo, B. Yang, F. Yin, J. S. A. Horng, S. Tian, Y. Fu, G. Xu, H. Deng, H. Lei, A. W. Tsen, K. Sun, R. He, L. Zhao, Observation of the polaronic character of excitons in a two-dimensional semiconducting magnet CrI_3. *Nat. Commun.* **11**, 4780 (2020).

8. J. Luo, X. Wang, S. Li, J. Liu, Y. Guo, G. Niu, L. Yao, Y. Fu, L. Gao, Q. Dong, C. Zhao, M. Leng, F. Ma, W. Liang, L. Wang, S. Jin, J. Han, L. Zhang, J. Etheridge, J. Wang, Y. Yan, E. H. Sargent, J. Tang, Efficient and stable emission of warm-white light from lead-free halide double perovskites. *Nature* **563**, 541–545 (2018).

9. K. Miyata, D. Meggiolaro, M. T. Trinh, P. P. Joshi, E. Mosconi, S. C. Jones, F. de Angelis, X.-Y. Zhu, Large polarons in lead halide perovskites. *Sci. Adv.* **3**, e1701217 (2017).

10. M. Kang, S. W. Jung, W. J. Shin, Y. Sohn, S. H. Ryu, T. K. Kim, M. Hoesch, K. S. Kim, Holstein polaron in a valley-degenerate two-dimensional semiconductor. *Nat. Mater.* **17**, 676–680 (2018).

11. L. M. Carneiro, S. K. Cushing, C. Liu, Y. Su, P. Yang, A. P. Alivisatos, S. R. Leone, Excitation-wavelength-dependent small polaron trapping of photoexcited carriers in α-Fe_2O_3. *Nat. Mater.* **16**, 819–825 (2017).

12. J. Kang, Y. S. Jung, S.-H. Wei, A. C. Dillon, Implications of the formation of small polarons in Li_2O_2 for Li-air batteries. *Phys. Rev. B* **85**, 035210 (2012).

13. M. Setvin, C. Franchini, X. Hao, M. Schmid, A. Janotti, M. Kaltak, C. G. van de Walle, G. Kresse, U. Diebold, Direct view at excess electrons in TiO_2 rutile and anatase. *Phys. Rev. Lett.* **113**, 086402 (2014).

14. Z. Wang, S. McKeown Walker, A. Tamai, Y. Wang, Z. Ristic, F. Y. Bruno, A. de la Torre, S. Riccò, N. C. Plumb, M. Shi, P. Hlawenka, J. Sánchez-Barriga, A. Varykhalov, T. K. Kim, M. Hoesch, P. D. C. King, W. Meevasana, U. Diebold, J. Mesot, B. Moritz, T. P. Devereaux, M. Radovic, F. Baumberger, Tailoring the nature and strength of electron-phonon interactions in the $SrTiO_3$(001) 2D electron liquid. *Nat. Mater.* **15**, 835–839 (2016).

15. S. Selcuk, A. Selloni, Facet-dependent trapping and dynamics of excess electrons at anatase TiO_2 surfaces and aqueous interfaces. *Nat. Mater.* **15**, 1107–1112 (2016).

16. T. Wang, C. Caraiani, G. W. Burg, W.-L. Chan, From two-dimensional electron gas to localized charge: Dynamics of polaron formation in organic semiconductors. *Phys. Rev. B* **91**, 041201 (2015).

17. S. Zhang, T. Wei, J. Guan, Q. Zhu, W. Qin, W. Wang, J. Zhang, E. W. Plummer, X. Zhu, Z. Zhang, J. Guo, Enhanced superconducting state in FeSe/$SrTiO_3$ by a dynamic interfacial polaron mechanism. *Phys. Rev. Lett.* **122**, 066802 (2019).

18. D. Emin, Large (bi)polarons for novel energy conversion and superconductivity. *J. Supercond. Nov. Magn.* **33**, 35–42 (2020).

19. F. Zuo, L. Wang, T. Wu, Z. Zhang, D. Borchardt, P. Feng, Self-doped Ti^{3+} enhanced photocatalyst for hydrogen production under visible light. *J. Am. Chem. Soc.* **132**, 11856–11857 (2010).

20. B. Li, J. Zhao, K. Onda, K. D. Jordan, J. Yang, H. Petek, Ultrafast interfacial proton-coupled electron transfer. *Science* **311**, 1436–1440 (2006).

21. X. Chi, L. Mandal, C. Liu, A. D. Fauzi, A. Chaudhuri, T. J. Whitcher, H. K. Jani, Z. Chen, S. Xi, C. Diao, M. A. Naradipa, X. Yu, P. Yang, A. H. Castro-Neto, M. B. H. Breese, K. P. Loh, T. V. Venkatesan, A. Rusydi, Unravelling a new many-body large-hole polaron in a transition metal oxide that promotes high photocatalytic activity. *NPG Asia Mater.* **14**, 19 (2022).

22. M. Dey, A. Singh, A. K. Singh, Formation of a small electron polaron in Tantalum oxynitride: Origin of low mobility. *J. Phys. Chem. C* **125**, 11548–11554 (2021).

23. Y. Natanzon, A. Azulay, Y. Amouyal, Evaluation of polaron transport in solids from first-principles. *Isr. J. Chem.* **60**, 768–786 (2020).

24. N. A. Deskins, M. Dupuis, Electron transport via polaron hopping in bulk TiO_2: A density functional theory characterization. *Phys. Rev. B* **75**, 195212 (2007).

25. A. J. Rettie, W. D. Chemelewski, D. Emin, C. B. Mullins, Unravelling small-polaron transport in metal oxide photoelectrodes. *J. Phys. Chem. Lett.* **7**, 471–479 (2016).

26. J. M. Garcia-Lastra, J. S. G. Myrdal, R. Christensen, K. S. Thygesen, T. Vegge, DFT+U study of polaronic conduction in Li_2O_2 and Li_2CO_3: Implications for Li–Air batteries. *J. Phys. Chem. C* **117**, 5568–5577 (2013).

27. A. de la Torre, D. M. Kennes, M. Claassen, S. Gerber, James W. McIver, M. A. Sentef, *Colloquium*: Nonthermal pathways to ultrafast control in quantum materials. *Rev. Mod. Phys.* **93**, 041002 (2021).

28. A. S. Disa, T. F. Nova, A. Cavalleri, Engineering crystal structures with light. *Nat. Phys.* **17**, 1087–1092 (2021).

29. E. Pastor, J.-S. Park, L. Steier, S. Kim, M. Grätzel, J. R. Durrant, A. Walsh, A. A. Bakulin, In situ observation of picosecond polaron self-localisation in α-Fe_2O_3 photoelectrochemical cells. *Nat. Commun.* **10**, 3962 (2019).

30. M. Park, A. J. Neukirch, S. E. Reyes-Lillo, M. Lai, S. R. Ellis, D. Dietze, J. B. Neaton, P. Yang, S. Tretiak, R. A. Mathies, Excited-state vibrational dynamics toward the polaron in methylammonium lead iodide perovskite. *Nat. Commun.* **9**, 2525 (2018).

31. B. Wu, W. Ning, Q. Xu, M. Manjappa, M. Feng, S. Ye, J. Fu, S. Lie, T. Yin, F. Wang, T. W. Goh, P. C. Harikesh, Y. K. E. Tay, Z. X. Shen, F. Huang, R. Singh, G. Zhou, F. Gao, T. C. Sum, Strong self-trapping by deformation potential limits photovoltaic performance in bismuth double perovskite. *Sci. Adv.* **7**, eabd3160 (2021).

32. C. Guo, X. Meng, H. Fu, Q. Wang, H. Wang, Y. Tian, J. Peng, R. Ma, Y. Weng, S. Meng, E. Wang, Y. Jiang, Probing nonequilibrium dynamics of photoexcited polarons on a metal-oxide surface with atomic precision. *Phys. Rev. Lett.* **124**, 206801 (2020).

33. Z. Jin, Y. Peng, Y. Fang, Z. Ye, Z. Fan, Z. Liu, X. Bao, H. Gao, W. Ren, J. Wu, G. Ma, Q. Chen, C. Zhang, A. V. Balakin, A. P. Shkurinov, Y. Zhu, S. Zhuang, Photoinduced large polaron transport and dynamics in organic-inorganic hybrid lead halide perovskite with terahertz probes. *Light Sci. Appl.* **11**, 209 (2022).

34. W. Zheng, B. Sun, D. Li, S. M. Gali, H. Zhang, S. Fu, L. di Virgilio, Z. Li, S. Yang, S. Zhou, D. Beljonne, M. Yu, X. Feng, H. I. Wang, M. Bonn, Band transport by large Fröhlich polarons in MXenes. *Nat. Phys.* **18**, 544–550 (2022).

35. S. Tang, W. Qiu, X. Xu, S. Xiao, Y. Tong, X. Wang, S. Yang, Harvesting of infrared part of sunlight to enhance polaron transport and solar water splitting. *Adv. Funct. Mater.* **32**, 2110284 (2022).

36. W. H. Sio, C. Verdi, S. Ponce, F. Giustino, Polarons from first principles, without supercells. *Phys. Rev. Lett.* **122**, 246403 (2019).

37. Z. Feng, V. Timoshevskii, A. Mauger, C. M. Julien, K. H. Bevan, K. Zaghib, Dynamics of polaron formation in Li_2O_2 from density functional perturbation theory. *Phys. Rev. B* **88**, 184302 (2013).

38. J. Lafuente-Bartolome, C. Lian, W. H. Sio, I. G. Gurtubay, A. Eiguren, F. Giustino, Ab initio self-consistent many-body theory of polarons at all couplings. *Phys. Rev. B* **106**, 075119 (2022).

39. M. Reticcioli, M. Setvin, M. Schmid, U. Diebold, C. Franchini, Formation and dynamics of small polarons on the rutile TiO_2(110) surface. *Phys. Rev. B* **98**, 045306 (2018).

40. P. M. Kowalski, M. F. Camellone, N. N. Nair, B. Meyer, D. Marx, Charge localization dynamics induced by oxygen vacancies on the TiO_2(110) surface. *Phys. Rev. Lett.* **105**, 146405 (2010).

41. D. Zhang, Z. K. Han, G. E. Murgida, M. V. Ganduglia-Pirovano, Y. Gao, Oxygen-vacancy dynamics and entanglement with polaron hopping at the reduced CeO_2 (111) surface. *Phys. Rev. Lett.* **122**, 096101 (2019).

42. W. H. Sio, C. Verdi, S. Poncé, F. Giustino, *Ab initio* theory of polarons: Formalism and applications. *Phys. Rev. B* **99**, 235139 (2019).

43. X. Tong, M. Bernardi, Toward precise simulations of the coupled ultrafast dynamics of electrons and atomic vibrations in materials. *Phys. Rev. Res.* **3**, 023072 (2021).

44. C. Aku-Leh, J. Zhao, R. Merlin, J. Menéndez, M. Cardona, Long-lived optical phonons in ZnO studied with impulsive stimulated Raman scattering. *Phys. Rev. B* **71**, 205211 (2005).

45. A. A. Maznev, F. Hofmann, A. Jandl, K. Esfarjani, M. T. Bulsara, E. A. Fitzgerald, G. Chen, K. A. Nelson, Lifetime of sub-THz coherent acoustic phonons in a GaAs-AlAs superlattice. *Appl. Phys. Lett.* **102**, 041901 (2013).

46. C. Verdi, F. Caruso, F. Giustino, Origin of the crossover from polarons to Fermi liquids in transition metal oxides. *Nat. Commun.* **8**, 15769 (2017).

47. J. G. Horstmann, H. Böckmann, B. Wit, F. Kurtz, G. Storeck, C. Ropers, Coherent control of a surface structural phase transition. *Nature* **583**, 232–236 (2020).

48. J. Cuffe, O. Ristow, E. Chávez, A. Shchepetov, P.-O. Chapuis, F. Alzina, M. Hettich, M. Prunnila, J. Ahopelto, T. Dekorsy, C. M. Sotomayor Torres, Lifetimes of confined acoustic phonons in ultrathin silicon membranes. *Phys. Rev. Lett.* **110**, 095503 (2013).

49. X. Meng, T. Pandey, J. Jeong, S. Fu, J. Yang, K. Chen, A. Singh, F. He, X. Xu, J. Zhou, W.-P. Hsieh, A. K. Singh, J.-F. Lin, Y. Wang, Thermal conductivity enhancement in MoS_2 under extreme strain. *Phys. Rev. Lett.* **122**, 155901 (2019).

50. J. Feng, C. Gong, H. Gao, W. Wen, Y. Gong, X. Jiang, B. Zhang, Y. Wu, Y. Wu, H. Fu, L. Jiang, X. Zhang, Single-crystalline layered metal-halide perovskite nanowires for ultrasensitive photodetectors. *Nat. Electron.* **1**, 404–410 (2018).

51. J. Xu, D. Chen, S. Meng, Decoupled ultrafast electronic and structural phase transitions in photoexcited monoclinic VO_2 *Sci. Adv.* **8**, eadd2392 (2022).

52. D. Lee, B. Chung, Y. Shi, G. Y. Kim, N. Campbell, F. Xue, K. Song, S.-Y. Choi, J. P. Podkaminer, T. H. Kim, P. J. Ryan, J.-W. Kim, T. R. Paudel, J.-H. Kang, J. W. Spinuzzi, D. A. Tenne, E. Y. Tsymbal, M. S. Rzchowski, L. Q. Chen, J. Lee, C. B. Eom, Isostructural metal-insulator transition in VO_2. *Science* **362**, 1037–1040 (2018).

53. N. Tancogne-Dejean, M. A. Sentef, A. Rubio, Ultrafast modification of Hubbard U in a strongly correlated material: Ab initio high-harmonic generation in NiO. *Phys. Rev. Lett.* **121**, 097402 (2018).

54. O. Gerbig, R. Merkle, J. Maier, Electron and ion transport in Li_2O_2. *Adv. Mater.* **25**, 3129–3133 (2013).

55. J. M. Soler, E. Artacho, J. D. Gale, A. García, J. Junquera, P. Ordejón, D. Sánchez-Portal, The SIESTA method for ab initio order-*N* materials simulation. *J. Phys. Condens. Matter* **14**, 2745–2779 (2002).

56. C. Lian, M. Guan, S. Hu, J. Zhang, S. Meng, Photoexcitation in solids: First-principles quantum simulations by real-time TDDFT. *Adv. Theory Simul.* **1**, 1800055 (2018).

57. P. You, D. Chen, C. Lian, C. Zhang, S. Meng, First-principles dynamics of photoexcited molecules and materials towards a quantum description. *WIREs Comput. Mol. Sci.* **11**, e1492 (2020).

58. J. Zhang, C. Lian, M. Guan, W. Ma, H. Fu, H. Guo, S. Meng, Photoexcitation induced quantum dynamics of charge density wave and emergence of a collective mode in 1*T*-TaS_2. *Nano Lett.* **19**, 6027–6034 (2019).

59. C. Lian, S.-J. Zhang, S.-Q. Hu, M.-X. Guan, S. Meng, Ultrafast charge ordering by self-amplified exciton-phonon dynamics in TiSe2. *Nat. Commun.* **11**, 43 (2020).

60. A. Einstein, *Investigations on the Theory of the Brownian Movement* (Dover Publications, 1956).

61. Z. Hu, W. Tan, S. Li, F. Pan, Charge transport mechanisms in potassium superoxide. *Phys. Chem. Chem. Phys.* **22**, 24480–24489 (2020).

62. M. D. Radin, D. J. Siegel, Charge transport in lithium peroxide: Relevance for rechargeable metal–air batteries. *Energ. Environ. Sci.* **6**, 2370–2379 (2013).

63. J. C. Tully, Mixed quantum–classical dynamics. *Faraday Discuss.* **110**, 407–419 (1998).

64. E. Runge, E. K. U. Gross, Density-functional theory for time-dependent systems. *Phys. Rev. Lett.* **52**, 997–1000 (1984).

65. M. Noda, S. A. Sato, Y. Hirokawa, M. Uemoto, T. Takeuchi, S. Yamada, A. Yamada, Y. Shinohara, M. Yamaguchi, K. Iida, I. Floss, T. Otobe, K.-M. Lee, K. Ishimura, T. Boku, G. F. Bertsch, K. Nobusada, K. Yabana, SALMON: Scalable ab-initio light–matter simulator for optics and nanoscience. *Comput. Phys. Commun.* **235**, 356–365 (2019).

66. N. Tancogne-Dejean, M. J. T. Oliveira, X. Andrade, H. Appel, C. H. Borca, G. le Breton, F. Buchholz, A. Castro, S. Corni, A. A. Correa, U. de Giovannini, A. Delgado, F. G. Eich, J. Flick, G. Gil, A. Gomez, N. Helbig, H. Hübener, R. Jestädt, J. Jornet-Somoza, A. H. Larsen, I. V. Lebedeva, M. Lüders, M. A. L. Marques, S. T. Ohlmann, S. Pipolo, M. Rampp, C. A. Rozzi, D. A. Strubbe, S. A. Sato, C. Schäfer, I. Theophilou, A. Welden, A. Rubio, Octopus, a computational framework for exploring light-driven phenomena and quantum dynamics in extended and finite systems. *J. Chem. Phys.* **152**, 124119 (2020).
